# Reduction of mutant ATXN1 rescues premature death in a conditional SCA1 mouse model

**DOI:** 10.1172/jci.insight.154442

**Published:** 2022-04-22

**Authors:** James P. Orengo, Larissa Nitschke, Meike E. van der Heijden, Nicholas A. Ciaburri, Harry T. Orr, Huda Y. Zoghbi

**Affiliations:** 1 Department of Neurology, Baylor College of Medicine, Houston, Texas, USA.; 2 Jan and Dan Duncan Neurological Research Institute at Texas Children’s Hospital, Houston, Texas, USA.; 3 Department of Neuroscience and; 4 Department of Molecular and Human Genetics, Baylor College of Medicine, Houston, Texas, USA.; 5 Department of Laboratory Medicine and Pathology, University of Minnesota, Minneapolis, Minnesota, USA.; 6 Department of Pediatrics, Baylor College of Medicine, Houston, Texas, USA.; 7 Howard Hughes Medical Institute, Houston, Texas, USA.

**Keywords:** Genetics, Neuroscience, Mouse models, Movement disorders, Neurodegeneration

## Abstract

Spinocerebellar ataxia type 1 (SCA1) is an adult-onset neurodegenerative disorder. As disease progresses, motor neurons are affected, and their dysfunction contributes toward the inability to maintain proper respiratory function, a major driving force for premature death in SCA1. To investigate the isolated role of motor neurons in SCA1, we created a conditional SCA1 (cSCA1) mouse model. This model suppresses expression of the pathogenic SCA1 allele with a floxed stop cassette. cSCA1 mice crossed to a ubiquitous Cre line recapitulate all the major features of the original SCA1 mouse model; however, they took twice as long to develop. We found that the cSCA1 mice produced less than half of the pathogenic protein compared with the unmodified SCA1 mice at 3 weeks of age. In contrast, restricted expression of the pathogenic SCA1 allele in motor neurons only led to a decreased distance traveled of mice in the open field assay and did not affect body weight or survival. We conclude that a 50% or greater reduction of the mutant protein has a dramatic effect on disease onset and progression; furthermore, we conclude that expression of polyglutamine-expanded ATXN1 at this level specifically in motor neurons is not sufficient to cause premature lethality.

## Introduction

Spinocerebellar ataxia type 1 (SCA1) is a progressive neurodegenerative disease with a typical age of onset in the 30s and initial symptoms consisting of cerebellar ataxia and poor motor coordination (1). This disease is caused by a trinucleotide (CAG) repeat expansion in the first coding exon of the gene *ATXN1* (2, 3). The pathogenic polyglutamine–expanded ATXN1 is more stable than its WT counterpart (4). This enhanced stability further promotes its association with native interactors, such as the transcriptional repressor Capicua (CIC) and is therefore thought to, at least in part, explain its toxicity (5, 6).

The bulk of investigation in SCA1 has focused on mechanisms leading to cerebellar degeneration, particularly the degeneration of Purkinje neurons; however, other noncerebellar symptoms such as muscle wasting, breathing and swallowing difficulties, stiffness, sensory abnormalities, and cognitive decline remain understudied (7–9). Of particular interest to us are the pathophysiological mechanisms that promote death in SCA1. Individuals with SCA1 prematurely die from respiratory complications, such as aspiration pneumonia. Weakness in the muscles supporting breathing and safe swallowing is an established major contributor to the development of aspiration pneumonia in a diverse array of neurological disorders. Motor neurons regulate these activities, and when they degenerate, such as in amyotrophic lateral sclerosis (ALS), individuals suffer from premature death by the same respiratory complications as those seen in SCA1 (10).

We have previously hypothesized that premature death in SCA1 is due to motor neuron degeneration (11). Several pieces of evidence support that motor neuron degeneration occurs in SCA1; this evidence includes clinical signs on neurological exams, electrophysiology data and autopsy descriptions (12–17). We and others previously published that there is motor neuron disease in a SCA1 mouse model (11, 18). Furthermore, we demonstrated that this degeneration is time locked to neuromuscular respiratory dysfunction in SCA1 mice (11). In this manuscript, we describe a potentially novel conditional SCA1 mouse model that we created to investigate whether expression of the pathogenic polyglutamine ATXN1 restricted to motor neurons is causative for muscle wasting, neuromuscular respiratory dysfunction, and premature death. We started by crossing this conditional SCA1 line to a ubiquitous Cre line and found that the F1 offspring of this cross recapitulate all the major SCA1 features. However, these mice produced about 50% less of the polyglutamine-expanded ATXN1, resulting in a significant delay in disease onset and progression. Next, we investigated the F1 offspring of our conditional SCA1 mice crossed with a motor neuron–specific Cre line, as well as Purkinje neuron– and skeletal muscle–specific Cre lines to assess the cell-specific effects on survival. For each of these cell-specific crosses, with the reduced polyglutamine-expanded ATXN1 levels produced in our conditional model, we did not recapitulate premature death. We conclude that pathology in more than 1 cell type may be necessary to drive premature death in SCA1.

## Results

### Generation of a conditional SCA1 mouse model.

**Figure 1:**
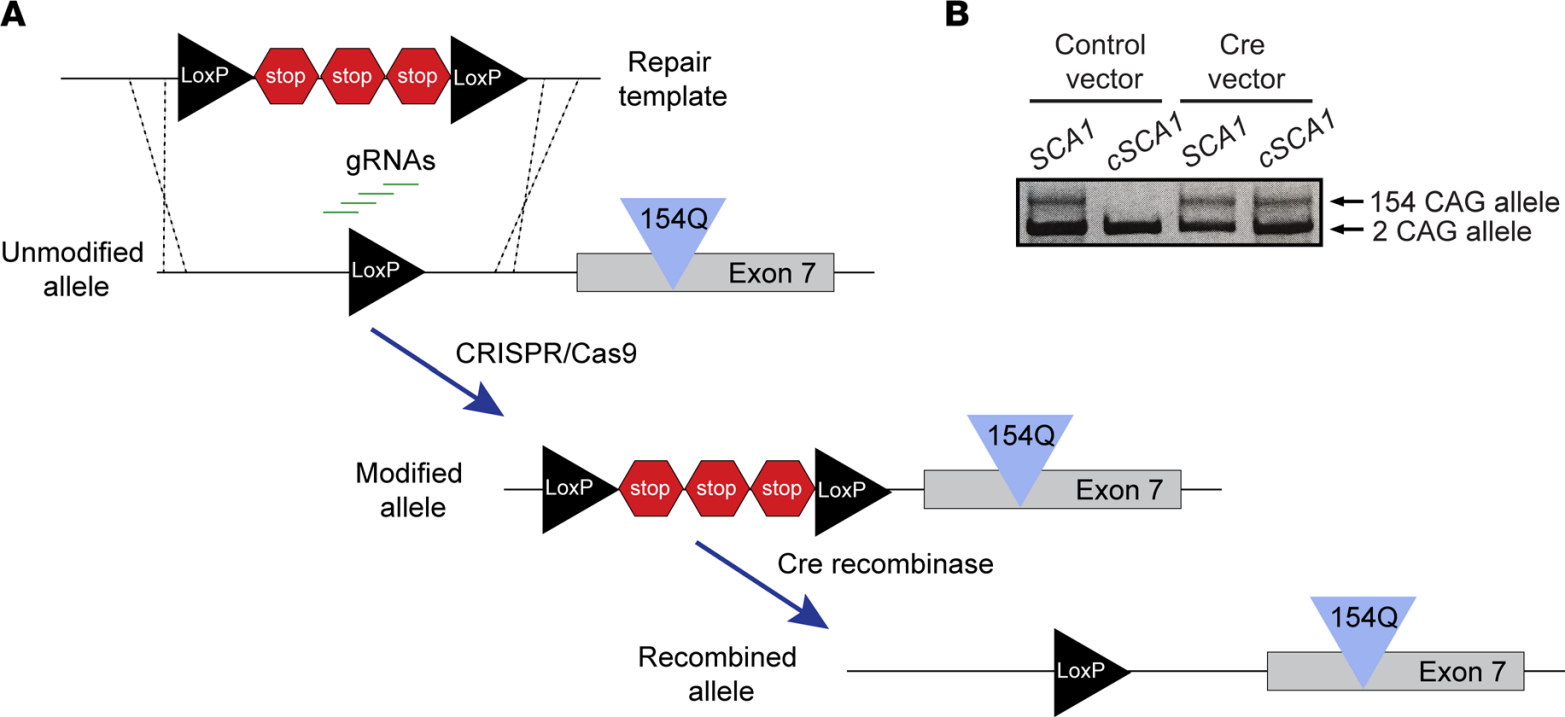
Generation of a conditional SCA1 mouse model. (**A**) Construct design illustrating the targeted location of the flox-stop-flox cassette knock-in using CRISPR/Cas9 tools. The location of the knock-in is in the intron upstream of the first coding exon (exon 7) in the endogenous mouse *Atxn1* gene. Specifically, the allele encoding the expanded CAG repeat tract was targeted for knock-in. (**B**) Reverse transcriptase PCR from primary fibroblast–derived *SCA1* and *cSCA1* mice. Cells were either electroporated with empty vector or a vector expressing Cre recombinase. *cSCA1* cells selectively expressed the expanded CAG repeat allele in the presence of Cre recombinase.

To investigate cell-specific contributions toward respiratory failure and premature death in SCA1, we set out to create a novel conditional mouse model, *Atxn1^154Q_flox_stop/+^* (hereafter referred to as *cSCA1*). With a conditional SCA1 mouse model, we can probe the effects of the pathogenic polyglutamine protein expression in a cell-specific manner. Our design focused on manipulating the well-established SCA1–knock-in mouse model, *Atxn1^154Q/+^* (hereafter referred to as *SCA1*) (19). To generate *cSCA1* mice, we utilized the CRISPR/Cas9 system to knock in a floxed stop cassette in the intron just upstream of the first coding exon of *Atxn1* (Figure 1A). Due to a unique sequence found in the upstream intron of the CAG repeat–containing allele, but not in the WT allele, we were able to specifically target the CAG repeat–containing allele. Using this approach, we were able to identify 1 founder mouse, a female. We confirmed the correct knock-in sequence and location by Sanger sequencing. To investigate if expression of the CAG repeat–expanded *Atxn1* allele was dependent on Cre recombinase, small clippings of the ear from our *cSCA1* founder mouse and a control unmodified *SCA1* mouse were collected, from which fibroblasts were extracted and grown in cell culture. These cultures were then electroporated with either a control vector or a vector expressing Cre recombinase. Forty-eight hours later, RNA was extracted from these cells, and reverse transcriptase PCR (RT-PCR) using primers that flank the CAG repeats was performed. The expected band size for WT *Atxn1* is 306 bp, while that for the CAG repeat *Atxn1* allele is 768 bp. We found that fibroblasts from the *cSCA1* mouse expressed only the WT *Atxn1* allele; however, when expressing Cre, it induced recombination and robust expression of the CAG repeat containing *Atxn1* transcript, similar to that of the *SCA1* mouse (Figure 1B). This established that our *cSCA1* mouse model effectively silenced expression of the pathogenic *Atxn1* allele in the absence of Cre recombinase. We next moved in vivo to validate that *cSCA1* mice crossed with a ubiquitous Cre driver produced polyglutamine-expanded ATXN1.

### cSCA1 mice produce less polyglutamine-expanded ATXN1 compared with SCA1 mice.

**Figure 2:**
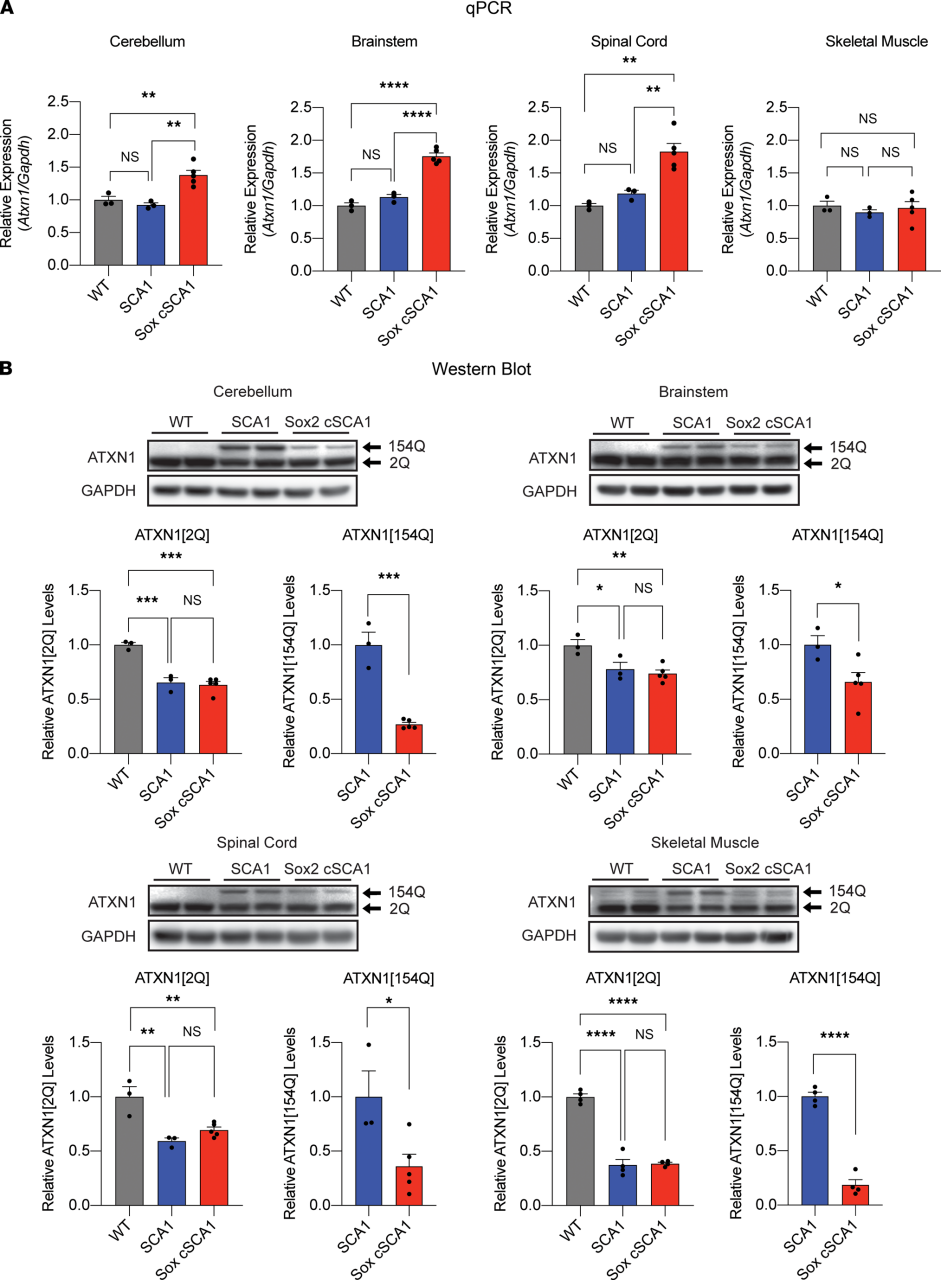
Mutant *Atxn1* mRNA and protein levels at 3 weeks of age. Tissue was collected from F1 progeny of *cSCA1* × *Sox2-Cre* mice at 3 weeks of age. Specifically, cerebellum, brainstem, spinal cord, and tibialis anterior skeletal muscle were harvested and divided in half, with one half being used to extract RNA and the other half for protein extraction. For each genotype (WT, *SCA1*, or *cSCA1 ×* *Sox2-Cre* [Sox2 cSCA1]), between 3 and 5 biological replicates were used. (**A**) qPCR was used to quantify mRNA expression of the *Atxn1* gene. Statistical assessment performed using 1-way ANOVA, followed by Tukey’s multiple comparisons test **P < 0.01 and ****P < 0.0001. (**B**) Western blot was utilized to quantify protein levels of either WT ATXN1(2Q) or the polyglutamine expanded ATXN1(154Q) using the 11750 antibody. A representative Western blot is provided for each tissue region, followed by the full quantification of all samples in the bar graph below. ATXN1(2Q) statistical assessment performed using 1-way ANOVA followed by Tukey’s multiple comparisons test *P < 0.05, **P < 0.01, ***P < 0.001, and ****P < 0.0001. ATXN1[154Q] statistical analysis was carried out with Student’s *t* test.

We crossed *cSCA1* mice to the ubiquitous Cre driver that expresses Cre recombinase by E6.5, *Sox2-Cre* mice (20). We verified that the *cSCA1 × Sox2-Cre* mice had complete recombination of the floxed allele and contained the same number of CAG repeats as the *SCA1* mice (Supplemental Figure 1; supplemental material available online with this article; https://doi.org/10.1172/jci.insight.154442DS1). Tissue was collected from WT, *SCA1*, and *cSCA1 × Sox2-Cre* mice at 3 weeks of age to quantitatively assess *Atxn1* RNA and protein levels. We chose to collect at this time point based on previously published data, together with our own unpublished findings demonstrating that this age yields the highest amount of soluble polyglutamine ATXN1 on Western blot in adult mouse brains (19). Quantitative PCR (qPCR) demonstrated a 2-fold increase in *Atxn1* mRNA levels in *cSCA1 × Sox2-Cre* mice, compared with WT or *SCA1* mice (Figure 2A). However, this difference was only seen in the cerebellum, brainstem, and spinal cord — and not in skeletal muscle tissue. To further isolate the pathogenic allele, we crossed these 3 genotypes with *Atxn1*-KO mice (21). We again observed the same pattern of mRNA expression seen when both the WT and expanded repeat alleles were expressed (Supplemental Figure 2), indicating that *cSCA1 × Sox2-Cre* express higher mRNA levels of the pathogenic allele than *SCA1* mice. At the protein level, however, the pattern flips. We observed that the pathogenic polyglutamine ATXN1 is expressed at half or less that amount in *cSCA1 × Sox2-Cre* mice compared with *SCA1* mice (Figure 2B). Assessing whole brain levels of polyglutamine ATXN1 in *cSCA1 × Sox2-Cre* compared with *SCA1* mice at P1 demonstrated a similar reduction as that seen at 3 weeks of age (Supplemental Figure 3). *Cic* is a transcriptional repressor and major protein interactor of ATXN1 (5). Previous literature has documented that CIC levels are unchanged in *SCA1* mice (6). We assessed CIC levels in whole brain tissue from age- and sex-matched controls, *SCA1,* and *cSCA1* × *Sox2-Cre* mice at P1 on Western blot. Despite the reduced levels of polyglutamine ATXN1 in *cSCA1* *×* *Sox2-Cre* mice, there was no significant difference in CIC levels between genotypes (Supplemental Figure 3). These studies demonstrate a significant reduction in levels of the polyglutamine-expanded ATXN1 in the *cSCA1* mice compared with *SCA1* mice. The inverse relationship at the mRNA level may represent a compensatory upregulation of the pathogenic allele, given its low protein expression (22).

### Spatial resolution in expression of pathogenic polyglutamine–expanded ATXN1.

**Figure 3:**
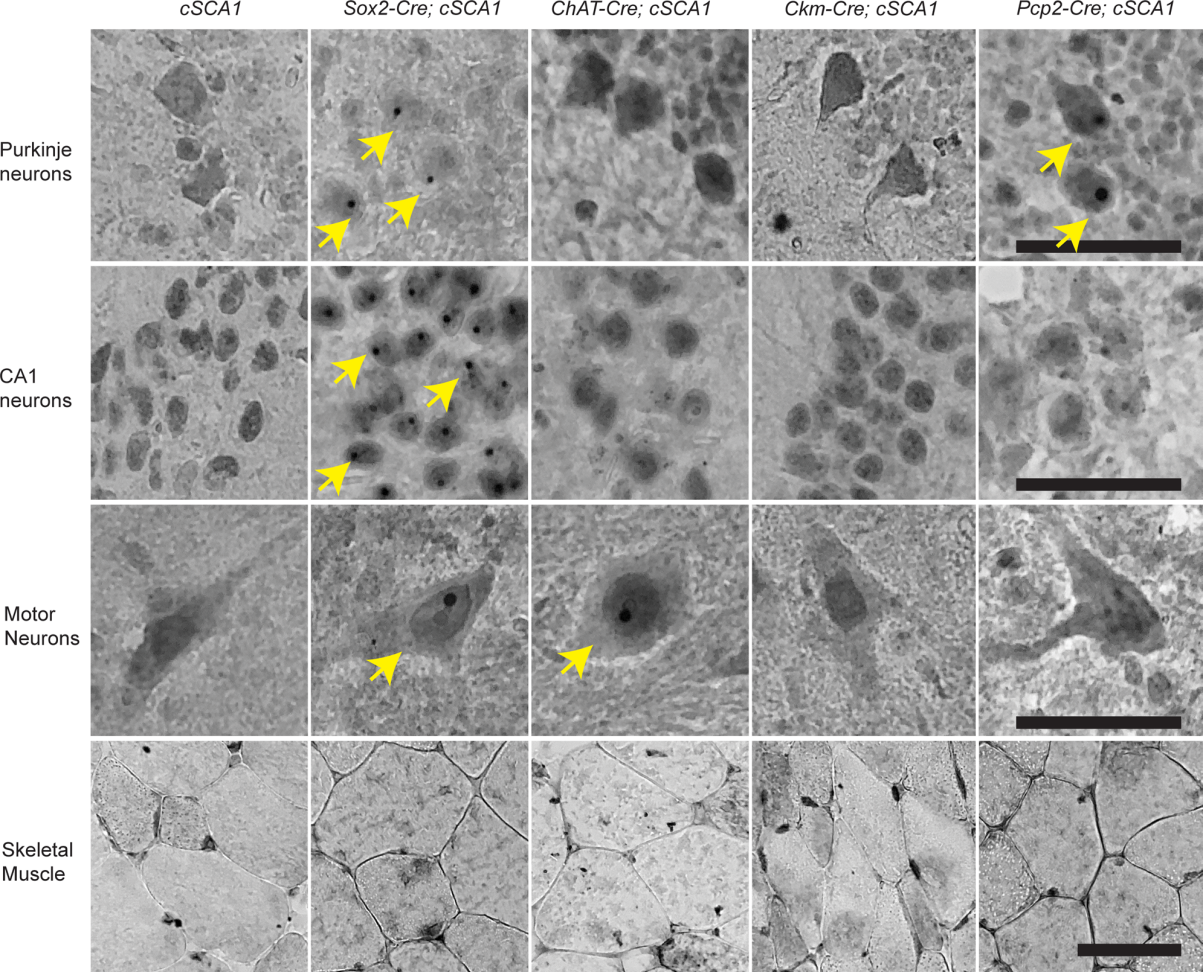
Cell type–specific restriction of the pathogenic polyglutamine–expanded ATXN1 protein defined by Cre driver. Tissue was harvested between 36 and 48 weeks of age from *cSCA1* mice and F1 offspring from *cSCA1* mice crossed with various Cre lines: *Sox2* (ubiquitous), *ChAT* (motor neuron), *Ckm* (skeletal muscle), and *Pcp2* (Purkinje neuron). Specifically, hippocampal CA1 neurons, cerebellar Purkinje neurons, cervical spinal cord motor neurons, and tibialis anterior skeletal muscle cells were imaged after staining with the ATXN1 11NQ antibody. Yellow arrows indicate nuclei with ATXN1 aggregate formation. Nuclear inclusions of ATXN1 protein occurs only in the presence of the polyglutamine expansion; thus, these inclusions are used as a proxy for expression of the recombined pathogenic allele. All images were taken with a 40× objective and then digitally zoomed to allow for optimal visualization of the nuclear inclusions. Scale bars: 50 μm.

Next, we assessed whether our *cSCA1* mice crossed with specific Cre drivers induced the polyglutamine-expanded ATXN1 exclusively in a cell type of interest, and we therefore crossed *cSCA1* mice to either a ubiquitous (*Sox2*), motor neuron–specific (*ChAT*), skeletal muscle–specific (*Ckm*), or Purkinje neuron–specific (*Pcp2*) Cre mouse line (20, 23–25). Purkinje neuron and skeletal muscle Cre drivers were chosen to address the alternative hypotheses that pathology within cell types other than motor neurons drives premature death. We surveyed for intranuclear ATXN1 aggregate formation in aged mice as a proxy for polyglutamine-expanded ATXN1 expression. Previous work demonstrated that nuclear aggregates of ATXN1 begin to form in motor neurons around 12 weeks of age and in Purkinje neurons around 24 weeks of age; therefore, we chose to use aged mice to ensure robust presence of aggregates (11, 19). We harvested whole brain, spinal cord, and skeletal muscle (tibialis anterior) tissue in mice between 36 and 48 weeks of age and performed IHC staining on sections of tissue using an ATXN1 polyclonal antibody (11NQ) (Figure 3). When we assessed CA1 hippocampal neurons, Purkinje neurons, and motor neurons, we found ATXN1 nuclear aggregates in all cell types in the F1 of *cSCA1 × Sox2-Cre*. As expected, we only detected aggregate formation in motor neurons in the *cSCA1 × ChAT-Cre* F1s. In the *cSCA1 × Pcp2-Cre* F1s, we saw intranuclear aggregates restricted to only the Purkinje neurons. Intriguingly, we were not able to detect intranuclear aggregates in skeletal muscle tissue from the F1s of *cSCA1* mice crossed with any of the Cre lines, including *Ckm-Cre*. Quantification of these results demonstrate that, in Purkinje neurons, 68% and 80% of the nuclei had ATXN1 aggregates for *cSCA1 × Pcp2-Cre* and *cSCA1 × Sox2-Cre* mice, respectively (Supplemental Figure 4), while 81% of hippocampal CA1 nuclei had aggregates in *cSCA1 × Sox2-Cre* mice. Furthermore, in motor neurons, 43% and 65% of the nuclei had ATXN1 aggregates for *cSCA1 × ChAT-Cre* and *cSCA1 × Sox2-Cre* mice, respectively (Supplemental Figure 4). 

As a complementary approach to confirm tissue specificity, we collected cerebellum, brainstem, spinal cord, and tibialis anterior skeletal muscle from the F1s of our *cSCA1* mice crossed with the above-mentioned Cre drivers to assess for polyglutamine-expanded ATXN1 via Western blot (Supplemental Figure 5A). *SCA1* mice displayed the appropriately sized band for the polyglutamine-expanded ATXN1 in all 4 tissues. As expected, there were reduced levels of the polyglutamine-expanded ATXN1 in the tissues from F1s of *cSCA1 × Sox2-Cre* mice. However, levels of the polyglutamine-expanded ATXN1 were undetectable in all tissues from F1s of our *cSCA1* mice crossed with either *ChAT-Cre, Ckm-Cre*, or *Pcp2-Cre*. Given that the *ChAT* and *Pcp2* Cre drivers turn on expression of the pathogenic allele in less than 5% of the total cell populations in these tissues, it’s not surprising that we found undetectable polyglutamine-expanded ATXN1 levels on Western blot. In muscle tissue, the predominate cell type is skeletal muscle; therefore, to confirm the pathogenic allele expression in this tissue from *cSCA1* mice crossed with either *Sox2-Cre* or *Ckm-Cre*, we performed RT-PCR (Supplemental Figure 5B). We have concluded that the detection of aggregated ATXN1 confirms the expression of the mutant allele in the neuronal cell types assessed and that the RT-PCR confirms its expression in skeletal muscle.

### Reduction of polyglutamine-expanded ATXN1 levels delays onset of muscle wasting, respiratory dysfunction, and lethality.

**Figure 4:**
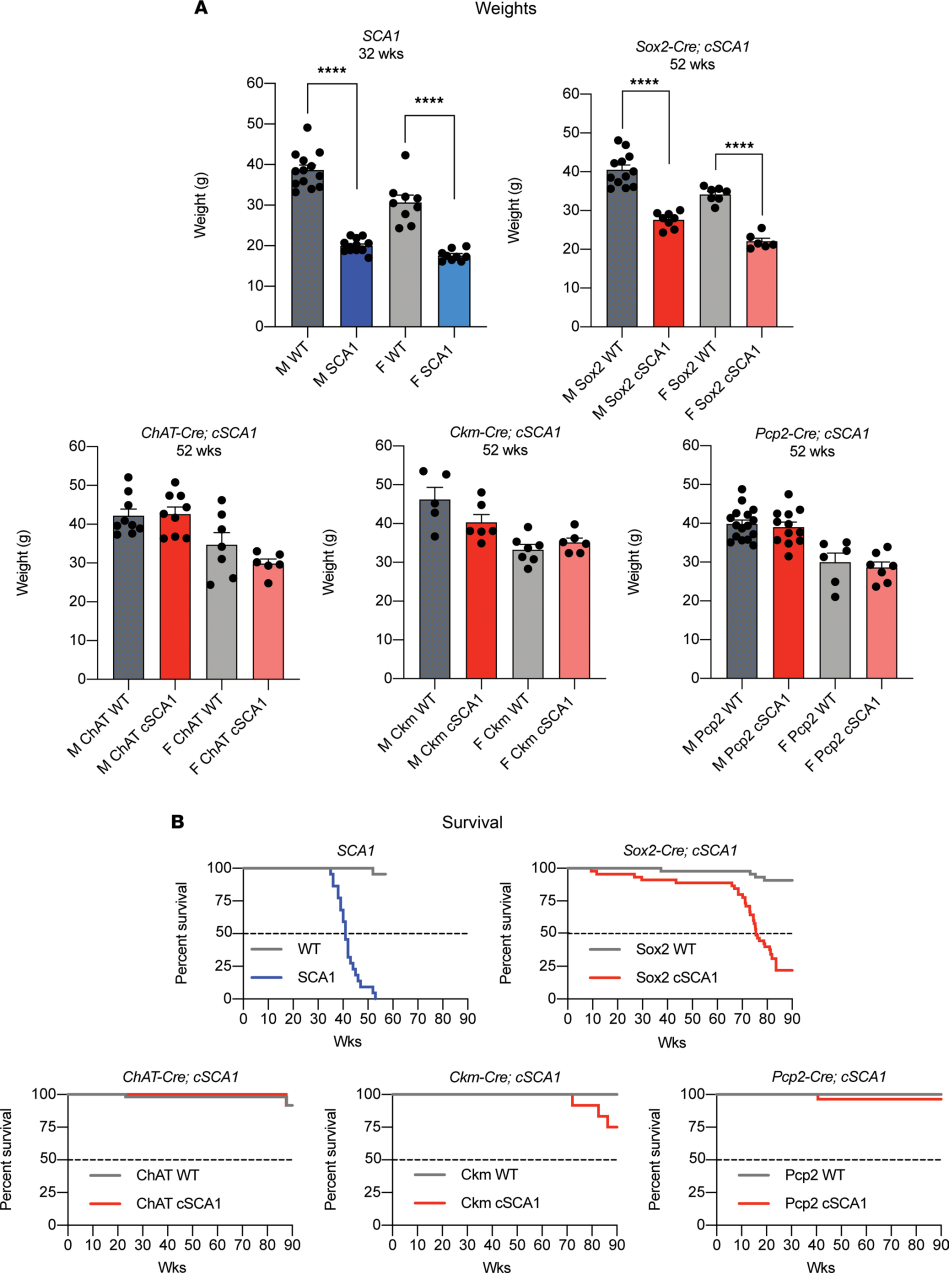
Cell-specific contributions of the polyglutamine-expanded ATXN1 protein on weight and lifespan. (**A**) Weights of *SCA1* mice at 32 weeks of age and F1 offspring of *cSCA1* mice crossed with various Cre lines: *Sox2* (ubiquitous), *ChAT* (motor neuron), *Ckm* (skeletal muscle), and *Pcp2* (Purkinje neuron) at 52 weeks of age. WT indicates either WT or Cre alone mice, *SCA1* indicates *Atxn1^154Q/+^* mice, and *cSCA1* indicates Cre × *Atxn1^154Q_flox_stop/+^* mice. “M” signifies males, and “F” signifies females. SCA1 group: M WT, *n =* 13, and F WT, *n =* 9; M SCA1, *n =* 13, and F SCA1, *n =* 9. Sox2 group: M WT, *n =* 12 and F WT, *n =* 7; M cSCA1 *n =* 8, and F cSCA1, *n =* 6. ChAT group: M WT, *n =* 9, and F WT, *n =* 7; M cSCA1, *n =* 9, and F cSCA1, *n =* 6. Ckm group: M WT, *n =* 5, and F WT, *n =* 7; M cSCA1, *n =* 6, and F cSCA1, *n =* 6. Pcp2 group: M WT, *n =* 16, and F WT, *n =* 6; M cSCA1, *n =* 12, and F cSCA1, *n =* 7. Student’s *t* test. ****P < 0.0001. (**B**) Survival as measured either at the time of natural death or humane euthanasia in *SCA1* and *cSCA1* mice crossed with various Cre lines. SCA1 group: WT, *n =* 22; SCA1, *n =* 22. Sox2 group: WT, *n =* 57; cSCA1, *n =* 49. ChAT group: WT, *n =* 57; cSCA1, *n =* 48. Ckm group: WT, *n =* 12; cSCA1, *n =* 12. Pcp2 group: WT, *n =* 49; cSCA1, *n =* 31.

Having established that *cSCA1* mice make less mutant protein from an early age, we wanted to know how this might affect survival and disease progression, especially within the context of the cell-specific lines. *SCA1* mice fail to gain weight beginning as early as 8 weeks of age, and this persists throughout their entire lives (19). In addition, these mice develop muscle wasting with neurogenic changes seen in their skeletal muscle, reduced lifespan, and neuromuscular respiratory weakness (11, 19). We aged *SCA1* mice and F1 mice from *cSCA1* mice crossed with the various Cre lines, and we measured their total body weight on a weekly basis. As expected, we found *SCA1* mice are significantly lighter than age- and sex-matched littermate controls at 32 weeks of age (Figure 4A). By 52 weeks of age, F1 mice, from *cSCA1 × Sox2-Cre* crosses, demonstrate a robust reduction in weight, as well (Figure 4A). The delay in weight loss can also be seen in the trend lines in Supplemental Figure 6, where *SCA1* mice begin diverging in their weights from controls around 12 weeks of age, while the F1 mice from *cSCA1 × Sox2-Cre* do so around 28 weeks of age. We did not see a significant difference in total body weight in F1 mice of crosses between *cSCA1* and either *ChAT-Cre, Ckm-Cre*, or *Pcp2-Cre* (Figure 4A and Supplemental Figure 6).

Individuals with SCA1 and *SCA1* mice both demonstrate reduced lifespans (1, 19). We measured survival as either the point in which mice spontaneously die or are requested to be humanely euthanized by our veterinarian staff to avoid unnecessary pain and suffering. Our colony of *SCA1* mice display a precipitous drop off in survival with a median survival of ~41 weeks of age (Figure 4B). Congruent with the total body weight data, we found a delay in premature death that was almost twice as long in the F1 mice of *cSCA1 × Sox2-Cre* mice (median survival ~75 weeks) compared with *SCA1*-alone mice (Figure 4B). F1 progeny from the *cSCA1* mice crossed with the various Cre lines (*ChAT-Cre, Ckm-Cre*, or *Pcp2-Cre*) failed to demonstrate significant premature death compared with their control littermates.

**Figure 5:**
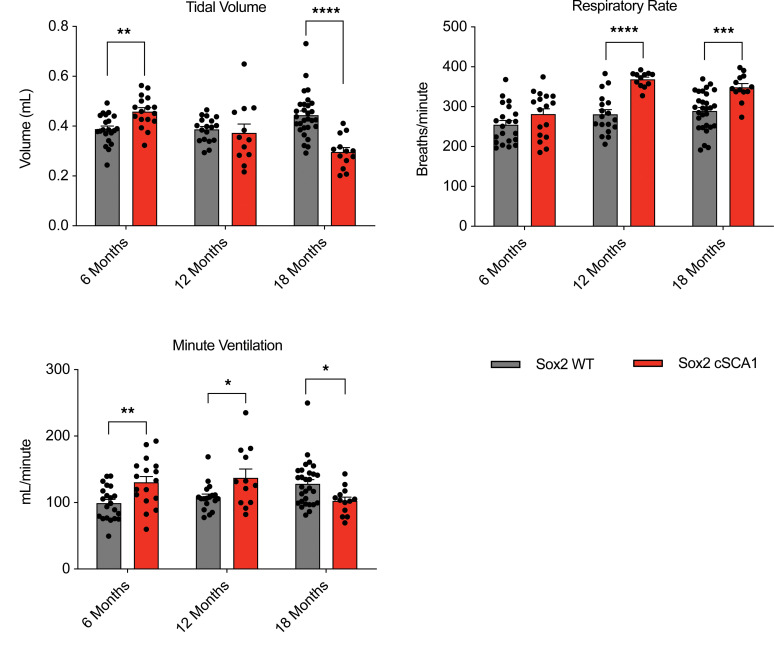
Neuromuscular respiratory failure in *cSCA1* mice crossed with *Sox2-Cre*. Whole-body plethysmography was performed on F1 progeny of *cSCA1* × *Sox2-Cre* mice at 6, 12, or 18 months of age. Sox2 WT, *n =* 30; Sox2 cSCA1, *n =* 15. Student’s *t* test. *P < 0.05, **P < 0.01, ***P < 0.001, and ****P < 0.0001.

We have previously linked decreased total body weight, skeletal muscle atrophy, and premature death with neuromuscular respiratory dysfunction around 24 weeks of age in *SCA1* mice using whole body plethysmography (11). Given that only the F1 mice from *cSCA1 × Sox2-Cre* demonstrate weight loss and premature death, we next wanted to determine whether they also manifest neuromuscular respiratory dysfunction. Indeed, close to the time of premature death at 72 weeks of age, and not earlier at 24 or 48 weeks of age, we found that these mice develop a significant reduction in tidal volume with a compensatory increase in respiratory rate, hallmarks of neuromuscular respiratory weakness (Figure 5). At the earliest time point, 24 weeks of age, we paradoxically found that *cSCA1 × Sox2-Cre* demonstrated a slight but significant increase in tidal volume compared with their age- and sex-matched littermate controls. In addition to respiratory muscle strength, there are other contributors to tidal volume, including elastic recoil of the lung, airway patency, and chest wall anatomy, and perhaps one of these factors accounts for this unexpected finding. We did not assess breathing physiology in the cell-specific Cre crosses, given that they failed to have a reduction in body weight and lifespan discussed above.

### Selective reduction of polyglutamine-expanded ATXN1 rescues motor coordination but not impaired locomotion.

**Figure 6:**
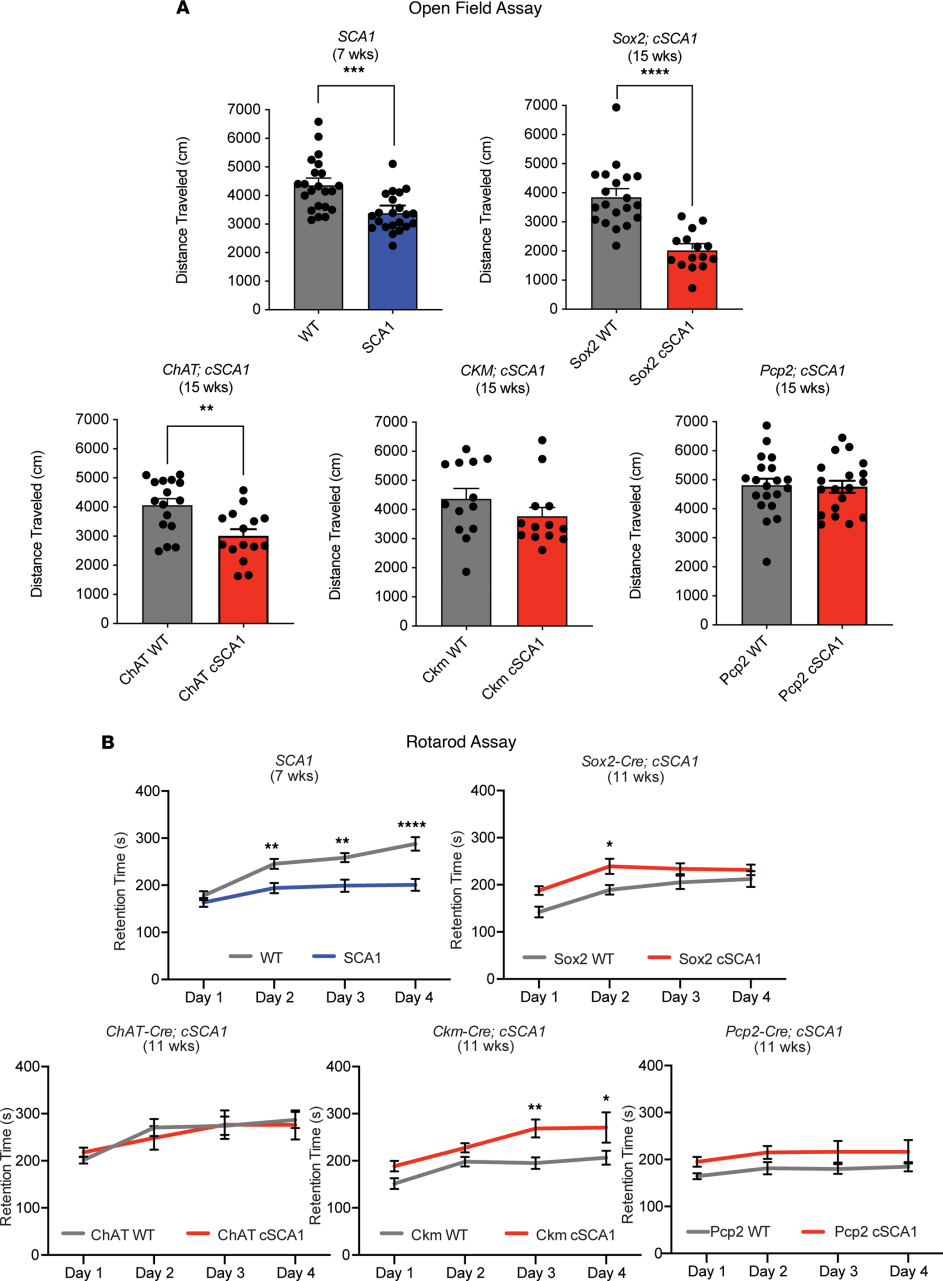
Behavioral effects of cell type–specific restriction of polyglutamine-expanded ATXN1 protein. (**A**) *SCA1* mice and F1 offspring of *cSCA1* mice crossed with various Cre lines: *Sox2* (ubiquitous), *ChAT* (motor neuron), *Ckm* (skeletal muscle), and *Pcp2* (Purkinje neuron) were subjected to open field assay at 7 or 15 weeks of age, respectively. Total distance traveled in cm during the assay was plotted. W) indicates either WT or Cre alone mice, and cSCA1 indicates Cre × *cSCA1* mice. *SCA1* group: WT, *n =* 22; SCA1, *n =* 22. ***P < 0.001 with *t* test. Sox2 group: WT, *n =* 20; cSCA1, *n =* 15. **** *P <* 0.0001 with *t* test. ChAT group: WT, *n =* 16; cSCA1, *n =* 15. **P < 0.01 with *t* test. Ckm group: WT, *n =* 13; cSCA1, *n =* 13. *P* > 0.05 with *t* test. Pcp2 group: WT, *n =* 20; cSCA1, *n =* 19. *P* > 0.05 with *t* test. (**B**) Retention time on an accelerating rotating rod was measured in *SCA1* and *cSCA1* mice crossed with various Cre lines at 7 or 11 weeks of age, respectively. *SCA1* group: WT, *n =* 22; SCA1, *n =* 22. Sox2 group: WT, *n =* 20; cSCA1, *n =* 16. ChAT group: WT, *n =* 17; cSCA1, *n =* 13. Ckm group: WT, *n =* 13; cSCA1, *n =* 13. Pcp2 group: WT, *n =* 16; cSCA1, *n =* 13. For each pair, a 2-way ANOVA was calculated with Sidak’s multiple comparisons test, *P < 0.05, **P < 0.01, and ****P < 0.0001.

To examine the effect of restricted pathogenic allele expression on motor function, we employed 2 behavioral assays, open field assay and rotarod assay. Open field assay entails placing a subject mouse in a chamber with sensors that measure how much and where the mouse moves within the chamber over a 30-minute time period (26). *SCA1* mice have a reduction in the overall distance traveled when assessed by the open field assay (Figure 6A). As expected, F1 mice from *cSCA1 × Sox2-Cre* cross demonstrated a similar reduction in distance traveled. Interestingly, we also found a significant reduction in the distance traveled in F1 mice from the *cSCA1 × ChAT-Cre* cross. F1 mice from the *cSCA1 × Ckm-Cre* or *cSCA1* × *Pcp2-Cre* crosses did not demonstrate any difference in distance traveled compared with their age- and sex-matched littermate controls. This implies that even the reduced expression of the pathogenic *Atxn1* allele, in motor neurons specifically, may be sufficient to induce a motor impairment phenotype of decreased distance traveled. While these reduced levels are not sufficient to cause this impairment in Purkinje neurons or skeletal muscle.

Next, we assessed for motor coordination using the rotarod assay. In this assay, mice are placed on a rotating rod that is gradually accelerating in speed, and the time on the rod before they fall off (retention time) is measured as a proxy of overall motor coordination (26). *SCA1* mice have been previously shown to have an impairment in their performance on the rotarod (19), and we independently confirmed this in our own cohort (Figure 6B). However, we did not find consistent or significant differences in rotarod performance in the F1 progeny of *cSCA1* mice crossed with the various Cre lines, compared with their specific age- and sex-matched littermate controls. In addition, we aged a cohort of the progeny from the *cSCA1 × Sox2-Cre* cross to 36 weeks of age and did not find a significant difference in their rotarod performance compared with controls (Supplemental Figure 7). One caveat to point out is that none of the 4 Cre-alone lines demonstrated the typical improvement over trials days in rotarod performance as shown by WT mice. Previously published work has demonstrated a “Cre effect” on other behavioral assays, and perhaps the expression of Cre is playing a role in our rotarod results (27). The lack of significant rotarod impairment in the F1 mice from *cSCA1 × Sox2-Cre* and *cSCA1 × Pcp2-Cre* crosses demonstrates that the reduction in pathogenic allele expression we found in our *cSCA1* mice is not adequate to trigger this cerebellar phenotype in SCA1.

## Discussion

Our investigation begins with creating a potentially novel tool, a conditional SCA1 (*cSCA1*) mouse model, that allows for cell-specific expression of the pathogenic polyglutamine–expanded ATXN1. By driving expression of the toxic polyglutamine–expanded ATXN1 in a specific cell type, we can test what role pathology in this cell type plays on the overall phenotype of disease. In this manuscript, we discuss the generation of our *cSCA1* mouse model and find that polyglutamine-expanded ATXN1 levels from the conditional mutant allele are reduced by more than half of those compared with the nonconditional polyglutamine–expanded ATXN1. While this finding was unexpected, we discovered, as the mice aged, that it allowed for a unique possibility: we could examine the effect of significantly reducing mutant ATXN1 levels on disease onset and progression.

*cSCA1* mice with ubiquitous expression of polyglutamine-expanded ATXN1 develop declining weight, neuromuscular respiratory dysfunction, and premature death twice as late as the *SCA1* mice. Furthermore, we found that this reduced level of toxic polyglutamine–expanded ATXN1 throughout the brain or specifically restricted in Purkinje neurons was not sufficient to produce a rotarod assay deficit. In contrast, this same reduced level of toxic protein in motor neurons was sufficient to drive a decreased distance traveled deficit in the open field assay. Importantly, when the *cSCA1* mice were crossed with a motor neuron Cre driver (*ChAT-Cre*), the offspring failed to develop weight loss and premature death, as they do in the case of ubiquitous expression of the pathogenic allele (*cSCA1 × Sox2-Cre)*. This could suggest that restricted expression of polyglutamine-expanded ATXN1 in motor neurons is not sufficient to drive these features of disease. An alternative explanation could be that the *ChAT-Cre* line has a lower recombination efficiency than the *Sox2-Cre* line; thereby, the former might manifest in even lower levels of polyglutamine-expanded ATXN1 in motor neurons than the latter, when crossed with *cSCA1* mice. We conclude from these findings that reduction of polyglutamine-expanded ATXN1 levels rescue many disease features of SCA1. This could be due to the degree of reduction we observed, selective reduction of the pathogenic allele while preserving the WT allele level, the fact that the reduction is present early in life, or a combination of all 3.

Prior attempts aimed at reducing ATXN1 levels in mice, such as antisense oligonucleotides (ASO), microRNA (miRNA), RNA interference (RNAi), or altering modifiers of ATXN1 have failed to yield as robust a rescue as we found — in particular, the approximately 34-week extension in lifespan (28–36). In some cases, the reduction of polyglutamine-expanded ATXN1 levels was less than the 50% reduction we found. In other cases, the intervention reduced polyglutamine-expanded ATXN1 levels later in life, compared with our reduction, which was as early as P1. A common theme all these prior interventions share is that they reduced both WT and polyglutamine-expanded ATXN1. In contrast, we have a reduction specifically in the polyglutamine-expanded ATXN1, leaving the WT protein unaffected.

We are actively investigating the mechanism by which *cSCA1* mice produce less polyglutamine-expanded ATXN1 compared with *SCA1* mice. Results from the F1 offspring generated by crossing *cSCA1* to *Sox2-Cre* mice refute the explanation that this is the result of decreased mRNA expression or incomplete recombination of the floxed stop cassette. While quantification of specific neuronal nuclei with ATXN1 aggregates in *cSCA1 × Sox2-Cre* mice ranges from 65% to 81%, we do not think this represents incomplete recombination; instead, it may represent a combination of the heterogenous formation of nuclear aggregates, together with the technical limitation of assessing images in 1 focal plane by microscopy. Sanger sequencing of the coding regions of *Atxn1* did not demonstrate any novel mutations arising during the generation of the cSCA1 line. However, sequencing of intronic regions did reveal a single base deletion in the intron upstream of the first coding exon of *Atxn1* (Supplemental Figure 8). This single nucleotide deletion is found in *cis* with the expanded repeat allele, unique to *cSCA1* mice, and not present in *SCA1* or WT mice. We are currently investigating a hypothesis that this mutation might reduce translation efficiency of polyQ ATXN1 via inclusion of a cryptic exon. Our findings suggest that the reduction of polyQ ATXN1 is at the level of protein regulation, either by affecting translation efficiency or stabilizing the nascent protein. By determining the precise mechanism for this reduction in toxic protein, we might learn important lessons that could be applied toward generating allele-specific therapies in SCA1.

Given the findings of this study, another point to consider is the role of misfolded polyglutamine-expanded proteins propagating through the brain. The prion model for neurodegeneration stipulates that misfolded protein aggregates are able to seed and self-propagate transcellularly to different regions of the brain as the disease course advances (37). Aged F1 offspring from our *cSCA1* mice crossed with cell-specific Cre lines demonstrated exquisite selectivity in the cell types that formed intranuclear ATXN1 aggregates. For example, when crossed with a Purkinje neuron Cre driver, we found typical intranuclear ATXN1 aggregates in Purkinje neurons, but no aggregates were found elsewhere in the brain, including within deep cerebellar nuclei to which Purkinje neurons project. This would argue against the ability of these protein aggregates to self-propagate throughout the nervous system. A caveat of this may be that the expression of polyglutamine-expanded ATXN1 in our model is low and that perhaps it is not sufficient to propagate aggregates to other neurons. However, we did find that the main driver of pathology was soluble protein levels of the polyglutamine-expanded ATXN1 and not the presence of intranuclear ATXN1 aggregates, in agreement with previous work (38).

Insight from this study strengthens our conceptualization of how pathology arises in SCA1. We propose that the extension of lifespan in the *cSCA1* mice is derived not only from expressing less than half the levels of polyglutamine-expanded ATXN1, but also expressing normal WT ATXN1 levels in cSCA1 mice. Additional support for this proposition comes from a prior study in which a serine residue (S776) critical for promoting ATXN1 protein stability after phosphorylation was mutated to an alanine. When this mutation was introduced specifically on the polyglutamine-expanded ATXN1, there was a partial rescue of SCA1 pathology (39). However, when both the WT and polyglutamine-expanded alleles contained the phospho-dead mutation at S776, the mice continued to develop characteristic SCA1 pathology at nearly the same pace as unmodified SCA1 mice (39). Another point of discussion our data raises, which is particularly relevant regarding therapeutic approaches, is the timing of reduced polyglutamine-expanded ATXN1 levels. Our cSCA1 mice presumably exhibit reduced levels of the toxic protein from conception, although the earliest we assessed levels in this manuscript was at P1. It’s not clear whether reduction of polyglutamine-expanded ATXN1 to a similar magnitude later in life would have the same protective effect. Determining the precise amount of reduction in polyQ ATXN1 needed to rescue each cell-specific phenotype, determining when this reduction is needed, and determining whether it needs to be mutant allele specific will be critical for the design of novel and effective gene-based therapies in SCA1 and — potentially — for the broader category of neurodegenerative proteinopathy-based diseases.

## Methods

### Mouse husbandry.

*SCA1* mice, whose generation and characterization were previously described (19), were backcrossed to C57BL/6J background for a minimum of 10 generations. All mouse lines were kept on a 12-hour light/dark cycle. The following commercial mouse lines were purchased from the Jackson Laboratory: B6.Cg-Edil3Tg(Sox2-cre)1Amc/J, B6;129S6-Chattm2(cre)Lowl/J, B6.FVB(129S4)-Tg(Ckmm-cre)5Khn/J, and B6.129-Tg(Pcp2-cre)2Mpin/J.

### Generation of cSCA1 mouse model.

*SCA1* mice were used to target the pathogenic CAG repeat allele with knock-in of a floxed stop cassette. Specifically, this cassette was inserted upstream of the first coding exon in *Atxn1*. We targeted sgRNAs to a small segment of unique sequence in this area only found on the CAG-expanded repeat allele and not on the WT allele. Using the “Guide Design Resources” website created by the Zhang lab (https://zlab.bio/guide-design-resources), we selected 4 of the top sgRNAs for the region of interest: regions 2, 8, 11, and 13 (Supplemental File 1). Fertilized embryos from *SCA1* mice were collected and microinjected with the mixture of sgRNAs, Cas9 protein, and our donor DNA using established techniques (40). The donor DNA was synthesized from GeneArts (Thermo Fisher Scientific) and contained 3 SV40 polyadenylation transcriptional termination signals concatemerized and floxed by loxP sites (Supplemental File 1). The design of the flox-stop-flox cassette was chosen from previous studies in the literature (41). *cSCA1* mice were backcrossed to C57BL/6J background for a minimum of 7 generations.

### Fibroblast culture, electroporation, and RT-PCR.

Primary fibroblasts derived from ear clippings from a sequence confirmed *cSCA1* founder mouse and an unmodified *SCA1* mouse. In brief, a clipping of ear tissue was washed in sterile phosphate buffer solution (PBS). Then, the tissue was transferred to a 1 mL solution of 0.25% Trypsin and 4 mg/mL collagenase type 1 (Worthington-Biochem, LS004196) and incubated for 1 hour at 37°C. During this incubation, the sample received 5 pulses of max speed vortex. At the conclusion of the incubation the ear tissue was triturated with a p1000 5 times, and then the entire solution was added to a 10 mm tissue culture plate with 9 mL of DMEM (containing 10% FBS and penicillin and streptomycin antibiotics). The next day, the ear tissue was removed from the plate and the media was exchanged. After 1 week of growth, cells were lifted off the plate with 0.25% Trypsin and replated in a 6-well tray after being electroporated (Nucleofector, Lonza) with either empty vector provided with Lonza kit or a vector expressing Cre recombinase. Forty-eight hours after electroporation, RNA was harvested from cells and DNase treated (TURBO DNA-free Kit Invitrogen, AM1907), and RT-PCR was performed using a primer pair flanking the CAG-expanded repeats 4291-FW PolyQ (5′-ACCTTCCAGTTCATTGGGTC-3′) and 4292-RV PolyQ (5′-GCTCTGTGGAGAGCTGGA-3′).

### Western blotting.

Homogenates of whole brain in P1 pups or cerebellar, brainstem, spinal cord, or tibialis anterior skeletal muscle tissue from 3-week-old mice (*n =* 3–5 per genotype) were prepared by Dounce homogenization in NETN buffer (100 mM NaCl, 20 mM Tris-HCl [pH 8.0], 0.5 mM EDTA, 1.5% NP-40, 1× Xpert Protease Inhibitor Cocktail from GenDEPOT and 1× Xpert Phosphatase Inhibitor Cocktail from GenDEPOT). Samples were sonicated for a total of 10 times, incubated for 30 minutes at 4°C, and spun at 11,000*g* at 4°C for 15 minutes. Protein concentrations of the supernatant were measured using the Pierce BCA Protein Assay Kit (Thermo Fisher Scientific). Samples were diluted and prepared in NuPAGE sample reducing agent (Invitrogen) and NuPAGE LDS Sample Buffer (Invitrogen). The samples were then boiled for 10 minutes and then run on NuPAGE 4%–12% Bis-Tris gel (1.5 mm, 15-well gels; Invitrogen) or NuPAGE 3%–8% Tris-Acetate Protein Gels (1.5 mm, 15-well gels; Invitrogen) in MES running buffer (50 mM MES, 50 mM Tris base, 0.1 % SDS, 1 mM EDTA). The proteins were subsequently transferred to Immobilon-FL PVDF membranes (Invitrogen, 0.45 μm). After blocking for 1 hour at room temperature with 1:1 Odyssey Blocking Buffer TBS (LI-COR Biosciences) in TBS, membranes were probed overnight at 4°C with anti-ATXN1 (11750VII in house, 1:2000; ref. 42), anti-GAPDH (MilliporeSigma, 6C5, 1:10,000), anti-Vinculin (Sigma-Aldrich, V9131, 1:5,000) or anti-Cic (1:1,000 in house; ref. 5) in 1:1 Odyssey Blocking Buffer and TBS (LI-COR Biosciences) in Tris-buffered saline (5 mM Tris [pH 7.5], 120 mM NaCl) with 0.1% Tween-20 (TBST). The secondary antibodies used were anti–rabbit IgG (H&L) DyLight 680–conjugated (Rockland Immunochemicals, 611-144-002, 1:10,000) and anti–mouse IgG (H&L) DyLight 800–conjugated (Rockland Immunochemicals, 610-145-002, 1:10,000) in 1:1 Odyssey Blocking Buffer and TBS (LI-COR Biosciences) in TBST. The membranes were washed 3 times with TBST and then imaged using the Odyssey CLx Imaging System (LI-COR Biosciences).

### qPCR.

RNA from cerebellum tissue was isolated from 3-week-old mice (*n =* 3–5 per genotype). Total RNA was isolated using TRIzol from Invitrogen following the manufacturer’s instructions. RNA was quantified using NanoDrop 1000 (Thermo Fisher Scientific), and random-primed cDNA was prepared from 1 μg total RNA using M-MLV Reverse Transcriptase (Invitrogen). qPCR was then performed with PowerUp SYBR Green Master Mix (Applied Biosystems), and samples were run on a real-time PCR detection system (Bio-Rad, CFX96). All samples were analyzed in triplicate, and *Atxn1* expression levels were normalized to the expression of the housekeeping gene *Gapdh*. Primers for *Gapdh* and *Atxn1* were obtained from MilliporeSigma. Primers used for *Gapdh* were Gapdh_FW (5′-AGGTCGGTGTGAACGGATTTG-3′) and Gapdh_RV (5′-TGTAGACCATGTAGTTGAGGTCA-3′), and primers used for *Atxn1* were Atxn1_FW (5′-GAGAATCGAGGAGAGCCAC-3′) and Atxn1_RV (5′- AGACTTCGACACTGACCTG-3′).

### IHC.

Mouse tissue was collected and fixed for 24 hours in 10% formalin. Standard techniques were used to embed tissue in paraffin and to take 10 μm sections in serial section every 100 μm. Sections were deparaffinized and rehydrated. Slides were treated with antigen retrieval by boiling for 9 minutes in 10 mM sodium citrate, 0.05% Tween 20, pH 6.0. The ATXN1 11NQ antibody, generated in the Zoghbi laboratory (38), was incubated at a concentration of 1:2000 for approximately 12 hours at 4°C. Primary antibody was detected with biotinylated goat anti–rabbit IgG (1:1000) (Jackson ImmunoResearch Laboratories) and visualized using an ABC reagent kit (Vector Laboratories) according to the manufacturer’s recommendations. All IHC experiments were performed in triplicate, and figures show representative results. Images were collected with an Echo Revolve light microscope. For quantification analysis, a minimum of 25 nuclei per mouse were counted within a region of interested determined by the anatomical location and morphology of the cells. The percentage of those cells with positive ATNX1 intranuclear aggregates was averaged from 3 mice per genotype, and then the mean ± SEM was plotted.

### Behavioral assays.

All behavioral tests were performed during the light phase of the 12-hour light/dark cycle by an experimenter blinded to the genotype of the mice. The mice had access to food and water ad libitum, except during the assay. All mice were age- and sex-matched within experiments, and littermate controls were used when possible. For each test, the mice were habituated for 30 minutes in the test room before testing. Testing was done at a room brightness of 200 lux with white noise playing at 60 dB. 

### Open field assay.

Seven- or 15-week-old mice were habituated for 30 minutes in the test room at 200 lux with white noise playing at 60 dB. Each mouse was placed singly in the clear Plexiglas box of the open field apparatus (OmniTech Electronics) and allowed to move freely for 30 minutes. Total distance traveled among other parameters were recorded using Fusion activity monitoring software. 

### Rotarod test.

The rotarod test was performed at 7 or 11 weeks of age to evaluate coordination and motor skill acquisition (type 7650; Ugo Basile). The mice were placed on the rotating rod (3 cm diameter, 30 cm long) for 4 trials every day for a period of 4 days. Each trial lasted a maximum of 10 minutes. The rod accelerated from 4 to 40 rpm in 5 minutes and remained at 40 rpm for the remaining 5 minutes. The amount of time it took for the mice to fall was recorded. Two subsequent rotations around the rod were also counted as a fall.

### Whole body plethysmography.

We measured the breathing phenotypes as previously described (11, 43). In short, mice were placed in Buxco whole-body plethysmography chambers and received fresh room at a rate of 0.5 L/min. Breath waveforms were recorded using Phonemah 3 software. We derived breathing parameters (tidal volume, frequency, and minute volume) using custom written MATLAB (Mathworks) code. We only included breaths recorded after the mice were able to habituate to the recording chambers for 1 hour. We reduced the inclusion of artifacts from sniffing or moving by excluding breaths that had an inspiratory time under 0.025 seconds, expiratory time over 10 seconds, or a 2-fold disparity between expiratory and inspiratory tidal volume. We further filtered out periods at which more than 10% breaths were taken at a frequency higher than 600 breaths/min. Only mice from which we recorded more than 150 reliable breaths were included in our statistical analysis. Statistical significance was determined using a 2-tailed *t* test at each time point.

### Statistics.

All data are represented as the mean ± SEM. When possible, experimental analysis was performed in a manner that blinded investigators to the genotype of the animal. Statistical tests were carried out in accordance with the experimental design. Two-group comparisons used Student’s 2-tailed *t* test, whereas multigroup comparisons used 1- or 2-way ANOVA. Survival analysis used log-rank test. In each case, *P <* 0.05 was considered statistically significant.

### Study approval.

Animal care and experimental procedures were approved by the IACUC of Baylor College of Medicine, according to NIH guidelines (*Guide for the Care and Use of Laboratory Animals*, National Academies Press, 2011).

## Author contributions

JPO designed, carried out, and analyzed experiments, as well as primarily wrote and edited the manuscript. LN performed and analyzed experiments, generated figures, and contributed to writing and editing the manuscript. MEVDH carried out and analyzed the plethysmography assay and contributed to writing and editing. NAC performed and contributed to the write up of the behavior assays. HTO contributed to editing this manuscript. HYZ contributed to experimental design and editing this manuscript.

## Supplementary Material

Supplemental data

## Version 1 03/15/2022

In-Press Preview

## Version 2 04/22/2022

Electronic publication
