# Supplementary material for: Reduction of mutant ATXN1 rescues premature death in a conditional SCA1 mouse model
Source: JCI Insight. 2022 Apr 22;7(8):e154442. doi: 10.1172/jci.insight.154442 (PMC9089789; doi:10.1172/jci.insight.154442)
Supplement: Supplemental data [file jciinsight-7-154442-s118.pdf]

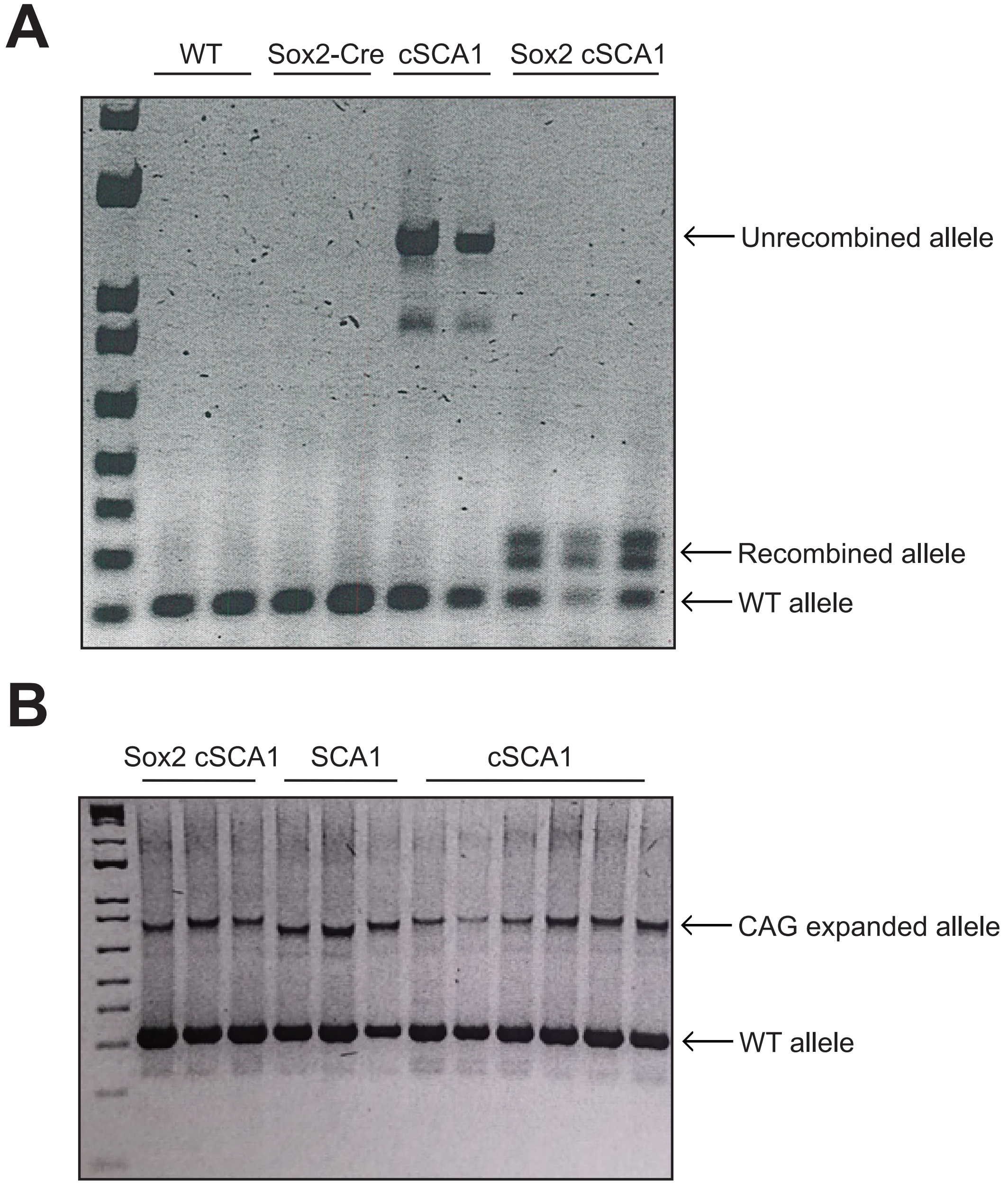

Supplemental Figure 1:
(**A**) DNA was extracted from whole brain tissue and polymerase chain reaction (PCR) was completed using primers that flank the flox-stop-flox cassette. Wildtype (WT) mice and Sox2-Cre alone mice contain the expected 210 bp band size for wildtype Atxn1 PCR product. cSCA1 alone mice produce a 210 bp WT band as expected plus the 1,234 bp unrecombined flox-stop-flox band. F1 offspring of cSCA1 crossed with Sox2-Cre demonstrate the WT 210 bp band plus the recombined mutant 348 bp band, which demonstrated 100% recombination of the flox-stop-flox cassette. (**B**) SCA1, cSCA1 alone and F1 offspring of cSCA1 crossed with Sox2-Cre mice had their DNA extracted from tail samples and PCR performed using a primer pair that flanks the CAG repeats. This PCR gives 2 products one which is 306 bp represents the wildtype allele and another which is approximately 768 bp represents the expand CAG repeat allele.

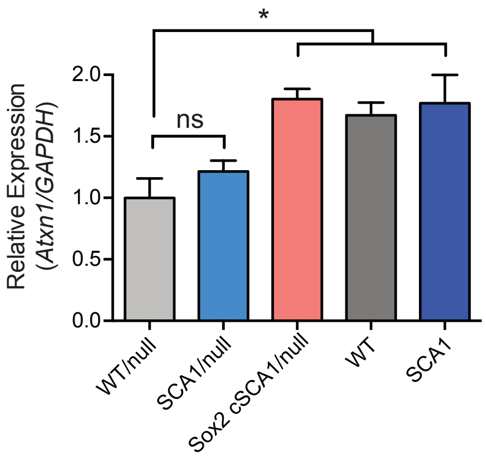

Supplemental Figure 2:
Wildtype (WT), Atxn1$^{154Q/+}$ (SCA1), and Atxn1$^{154Q_flox_stop/+}$, Sox2-Cre (Sox2 cSCA1) mice were crossed with Atxn1 knock-out mice to achieve lines with one allele of Atxn1 isolated as indicated by (/null). As controls mice with two copies of Atxn1 (WT or SCA1) were also used. Five mice per genotype were used a biological replicates and cerebellar tissue was dissected out at 5 weeks of age to extract RNA. Quantitative realtime polymerase chain reaction was used to quantify mRNA expression of the Atxn1 gene. Student's T test (*) signifies $p < 0.05$.

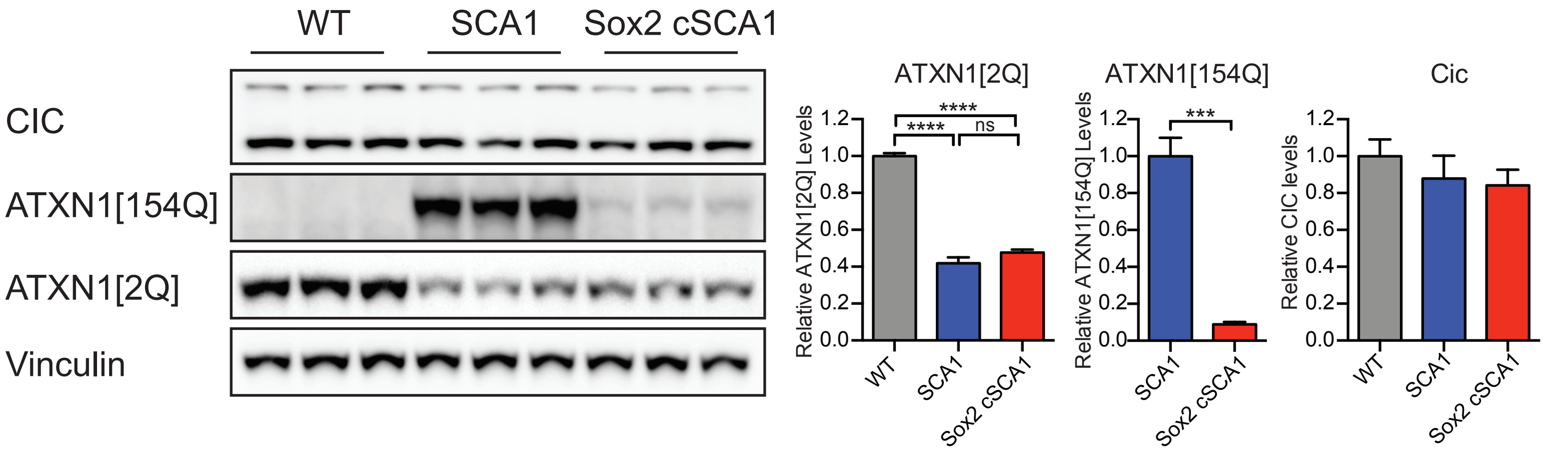

Supplemental Figure 3:
Whole brain was collected at post-natal day 1 from either wildtype (WT), $Atxn1^{154Q/+}$ (SCA1), or F1 progeny of $Atxn1^{154Q\_flox\_stop/+}$ crossed with Sox2-Cre mice (Sox2 cSCA1). Western blot was utilized to detect protein levels of either WT ATXN1[2Q] or the polyglutamine-expanded ATXN1[154Q] and Capicua protein levels (CIC). Vinculin protein levels were used as the loading control. Bar graphs represent the mean of 3 biological replicates +/- SEM. Student's T test (*) signifies p <0.05, (**) signifies p <0.01, (***) signifies p <0.001, and (****) signifies p <0.0001.

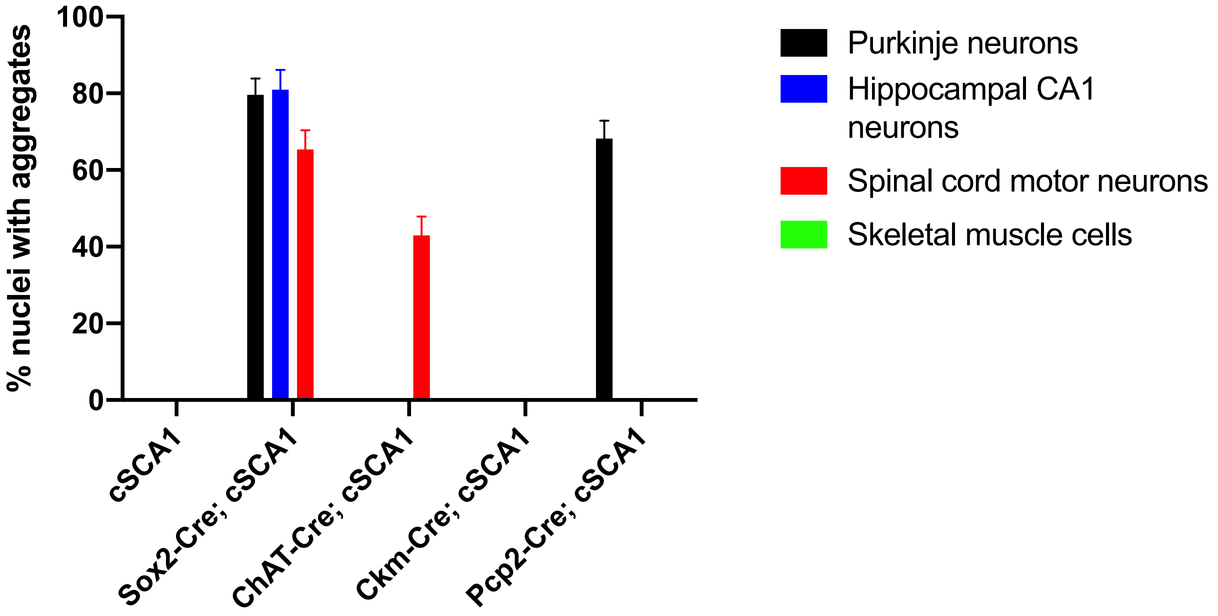

Supplemental Figure 4:
Quantification of neuronal nuclei with ATXN1 aggregates, representative images displayed in Figure 3. Within brain tissue specific neuronal cell populations were identified (Purkinje, CA1 or motor neurons) based on anatomical location and morphology. 3 mice per genotype were assessed and for each mouse a minimum of 25 nuclei per section were counted. The percentage of nuclei containing ATXN1 aggregates was quantified relative to the total number of nuclei assessed. For Purkinje neurons in Sox2-Cre; cSCA1 mice the mean was 79.6% (std dev 7.4), and for Pcp2-Cre; cSCA1 it was 68.2% (std dev 8.1). For CA1 hippocampal neurons in Sox2-Cre; cSCA1 mice the mean was 81.0% (std dev 8.9). For motor neurons in Sox2-Cre; cSCA1 mice the mean was 65.4% (std dev 8.8), and in ChAT-Cre; cSCA1 mice it was 42.9% (std dev 8.6). For all groups without a bar graph the value of neurons with aggregates as a percentage of total neuronal nuclei counted was zero.

A

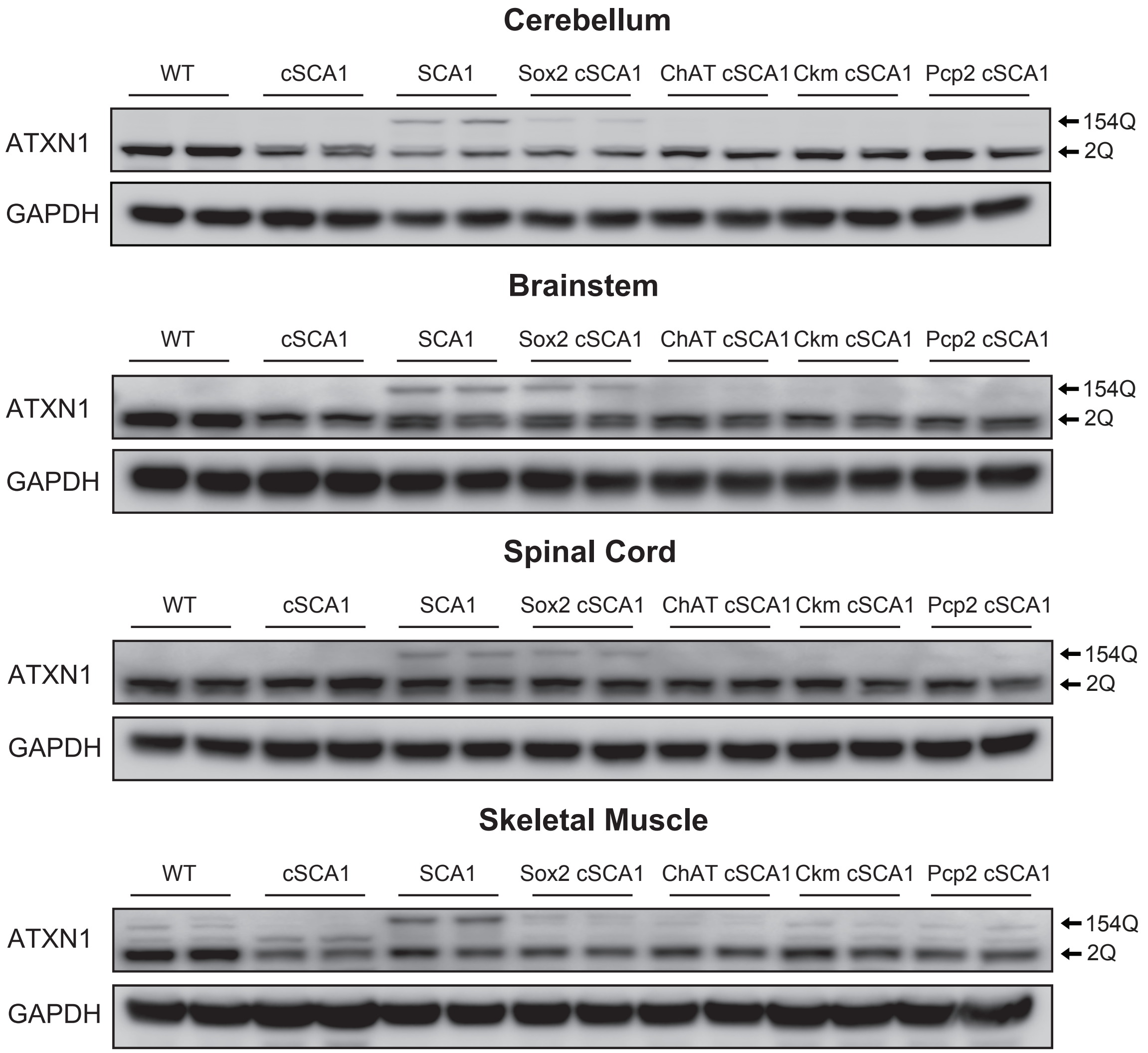

B

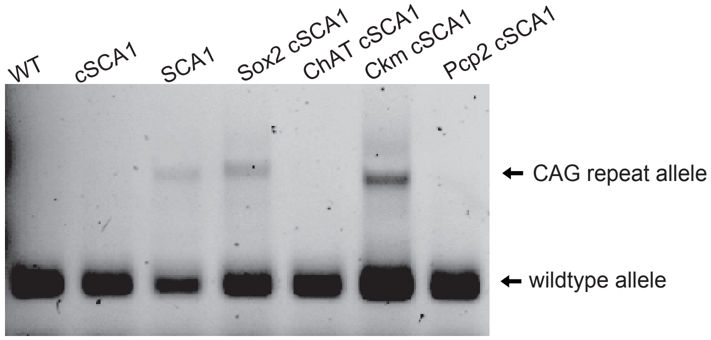

Supplemental Figure 5:

(**A**) Tissue was collected at 3 weeks of age from F1 progeny of either wildtype (WT), Atxn1$^{154Q_flox_stop/+}$ (cSCA1), Atxn1$^{154Q/+}$ (SCA1), or F1 progeny of Atxn1$^{154Q_flox_stop/+}$ (cSCA1) crossed with various Cre lines: Sox2, ChAT, Ckm or Pcp2. Western blot was utilized to detect protein levels of either WT ATXN1[2Q] or the polyglutamine-expanded ATXN1[154Q] using the 11750 antibody. (**B**) RNA was extracted from tibialis anterior skeletal muscle tissue at 3 weeks of age for the same 7 genotypes presented in panel A. The RNA was then DNAse treated to remove genomic DNA contamination followed by reverse transcription for cDNA synthesis. PCR was used with a primer pair (4291-FW PolyQ and 4292-RV PolyQ) that amplifies the region spanning the CAG repeats. This PCR yields two bands: a 768 base pair band that represents the expanded CAG repeat allele and a 292 bp band that represents the wildtype allele. In skeletal muscle the expanded CAG allele is expressed in SCA1 mice, and cSCA1 mice that are crossed with either Sox2-Cre or Ckm-Cre.

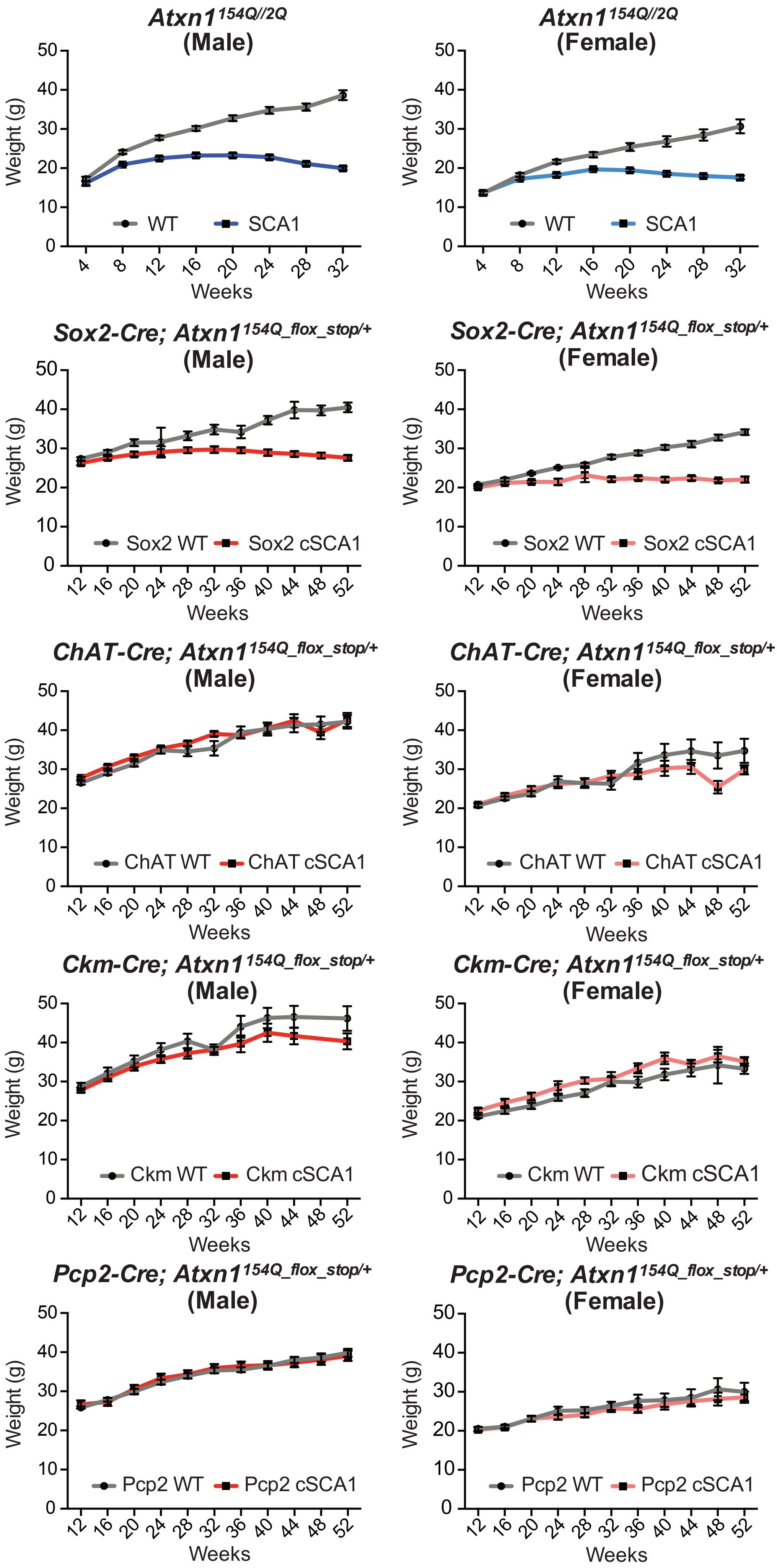

Supplemental Figure 6:
Weekly weights were measure in the $Atxn1^{154Q/+}$ (SCA1) or the $Atxn1^{154Q\_flox\_stop/+}$ (cSCA1) crossed with various Cre lines: Sox2, ChAT, Ckm or Pcp2. Age and sex matched littermates were included as controls (WT) being either wildtype or Cre alone as specified in graph. $Atxn1^{154Q/+}$: M WT n = 13, F WT n = 9, M SCA1 n = 13 and F SCA1 n = 9. Sox2 group: M WT n = 12, F WT n = 7, M cSCA1 n = 8 and F cSCA1 n = 6. ChAT group: M WT n = 9, F WT n = 7, M cSCA1 n = 9 and F cSCA1 n = 6. Ckm group: M WT n = 5, F WT n = 7, M cSCA1 n = 6 and F cSCA1 n = 6. Pcp2 group: M WT n = 16, F WT n = 6, M cSCA1 n = 12 and F cSCA1 n = 7.

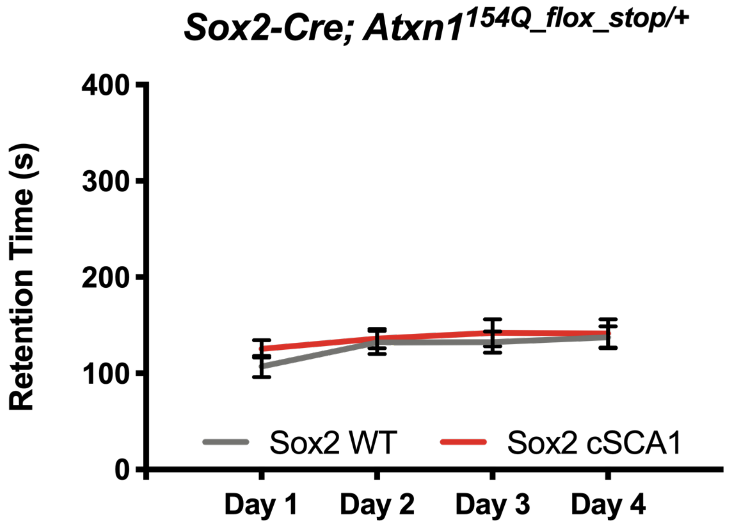

Supplemental Figure 7:
Retention time on an accelerating rotating rod was measured in Atxn1$^{154Q_flox_stop/+}$ mice crossed with Sox2-Cre (Sox KI n=17), along with age and sex matched littermate controls (WT n=17) at 36 weeks of age. Data points represent mean +/- SEM. Student’s T test demonstrates no statistical difference between genotypes.

Atxn1 intronic variant identified in cSCA1 mice

Location: chromosome 13 [45590211] TG>T

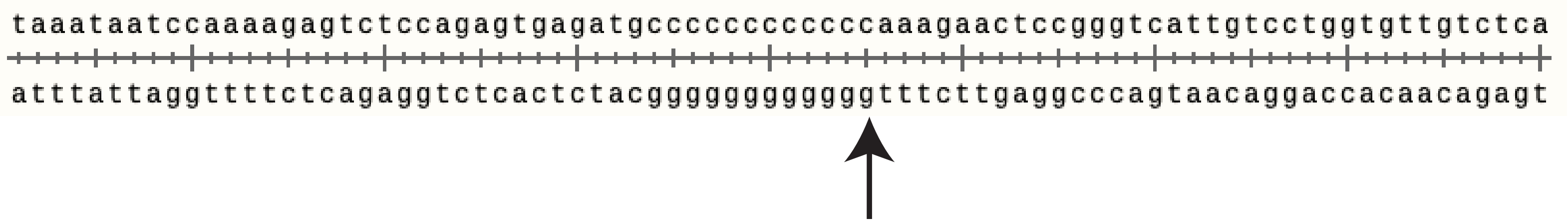

Single G nucleotide deletion

To scale diagram of mutation location in the *Atxn1* gene

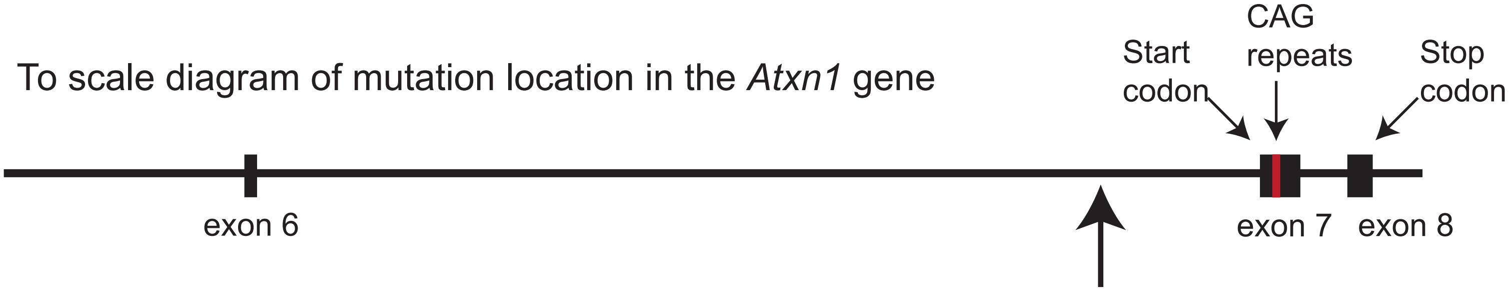

Single G nucleotide deletion

Supplemental Figure 8:
Sanger sequencing of the intronic region just upstream of the first coding exon in *Atxn1* revealed a single G nucleotide deletion in cis with the CAG repeat expanded allele. This deletion is only found in cSCA1 mice and not SCA1 or wildtype mice.

Supplemental file 1

<u>sgRNA #2</u>: ATATCGAATTCGAGCTCGCCCGG

<u>sgRNA #11</u>: CGAAGTTATATTAAGGGTTCCGG

<u>sgRNA #13</u>: GTATGCTATACGAAGTTATTAGG

<u>sgRNA #8</u>: TCCCCCGGGCTGCAGGTCGAGGG

<u>DNA donor sequence</u>
ctcgagttagagttttcattttgatttgtttttttttgtttgtttgtgtgtttctttgtttgtttgcctgtttgcttgcttactttttctgcc
cctttagacagggtctttctttgtagcccaagcagctggtctacaaagtctcaactttcctgcctcagcctcccatgggctgagat
tacagacactcgtgaccacacccagtttgagggtctgttctaaagtcagatcataggaaaaacaatctgaggaggaaatgtat
tcattcaagttccaaccccagaaaagcagctccccagatcagagcctttgatgttgtttccctctgcagcttcaccgtgtgggca
gtgctcctatctcctgctgctaccgtgcttcacactggtcttcttggcagtctaccctgtgccaggtagacattgctcacacaagg
gtggtgactcagaattcagtgtctctctgagatggaggcagggattgttcccgtaagagttcctggcatcgctaggtttggaca
cgtggctgcaatttgtctggcaaatatttcatcccctgctcatagtgagcaggtccctgctggggagaagaggaggagatgtg
gagagaaaatattcagagacaaacttggaagagaaggcacgtgggtgaagcacgtgcctaacttttattaaggaaactttca
aagcctaggttcaactttactttcatattcatgtacttatttatgcagcatttttatgagaccgcattttatactgtagcacaagga
agcctaaagttgtggtcattttcctgccccagttttcaaagtgctggaattacatgagccactataacagggtttatcttacatat
tatgtttggtttaataaaaaaaaaactagaatagtttttgtaactatctcaaagtcgtcttactgtttccttataagtgactgtac
atgaaattcccttgagacttatagtgagaagaattattttgggatactgctgctgagttggggtacatgggtgtattgtgggag
gcactgttagaataggggacactgctgtagattgagtgtagccctgaagccaagtgagtttgggtctggcatcaaagctgccc
aaagactcactgggcatctctattcaaaacttgctccttcacaaaacaattgggatcttattggactgagataaacttactcaa
atgacggggaatggggctccGTACCATAACTTCGTATAatgtatgcTATACGAAGTTATccTctagaACTAGT
GgatcTGCTGCACGTCTAGGGCGcagtagtccagggtttccttgatgatgtcatacttatcctgtcccttttttttccaca
gCTCGCGGTTGAGGACAAACTCTTCGCGGTCTTTCCAGTGGGGATCTGCGACTCTAGAGGATCT
GCGACTCTAGAGGATCataatcagccataccacatttgtagaggttttacttgctttaaaaaacctcccacacctccccc
tgaacctgaaacataaaatgaatgcaattgttgttgttaacttgtttattgcagcttataatggttacaAATAAAgcaatagc
atcacaaatttcacaaataaagcatttttttcactgcattctagttgtggtttgtccaaactcatcaatgtatcttatcatgtctG
GATCTGCGACTCTAGAGGATCataatcagccataccacatttgtagaggttttacttgctttaaaaaacctcccacac
ctcccccctgaacctgaaacataaaatgaatgcaattgttgttgttaacttgtttattgcagcttataatggttacaaataaagca
atagcatcacaaatttcacaaataaagcatttttttcactgcattctagttgtggtttgtccaaactcatcaatgtatcttatcatg
tctGGATCTGCGACTCTAGAggatcataatcagccataccacatttgtagaggttttacttgctttaaaaaacctcccac
acctcccccctgaacctgaaacataaaatgaatgcaattgttgttgttaacttgtttattgcagcttataatggttacaaataaag
caatagcatcacaaatttcacaaataaagcatttttttcactgcattctagttgtggtttgtccaaactcatcaatgtatcttatca
tgtctGGATCCATAACTTCGTATAatgtatgcTATACGAAGTTATccAGGTACcgatcccccgggctgaccaa
cctttctgtgtaaacaatttacagaaatgcatcatctaccaaaaaattgggggggggggctgtgatcctaacttttgggaggct
gatgcaggaggatcatgaattcaagccagccttcattgcatggccaatttgaggccagcctgagcaacaagagatgctgttaa
aaaaaaaaaaaaaaaaaagtgaccatccaccctccaggaagagaagaaaggggtagaggagagagaagaggcagaga
aatgtttgagttgctcagttgcctgctctattttgttaagaaagtgcatctgtgcaggagacgtggccggtggtcaagacagccg
cttactgctcttctagaggatctaagttccggtcctagcactcacactgtgattcacagctgccaataactccagtgtcaggaga

cccaatgccctcttgtggtctctgtaggcactggcacataggtggtgtgcataaactctcacaagcacctgcgcaaataaataa
atcataagtaagcaaatgtttaagaaacttagtcactattactggctctttagaccaggtaacattttatctattttaacacttag
gccataatctgcttttggaattccaactgagagggtccctctcctcttagtcttgttggtaatagtgtttttggtggttggtgggtt
gattggttggtttggcttggtggggtttctctccctagagctgacgttttgatttttttcccccctgtcttctctctttcctttcctcatc
tcctcctcctcctcctcctcctcctcctcctcctcctcagaatccggagctgccactgccagcctaaagaacccacggggag
atgattccccatgaagggcctggatcccctacagaaatccaatgtgactctctgtttatcagactaaaaccagagccggccag
ccagtgaaacagccaccgtggaggggggacggcgaaaaATGAAATCCAACCAAGAGCGGAGCAACGAATG
CCTGCCTCCCAAGAAACGTGAGATCCCCGCCACCAGCCGGCCCTCGGAGGAGAAGGCCACTGC
TCTGCCCAGCGACAACCACTGCGTGGAGGGTGTGGCCTGGCTCCCCAGCACCCCTGGCATCCGC
GGCCATGGGGGTGGGCGGCACGGGTCAGCAGGGACTTCCGGGGAGCATGGTTTACAAGGAAT
GGGTTTAaagctt

Supplemental file 1:
Guide RNA sequences and donor DNA sequence.
